# Diaqua­bis­(2-chloro­benzoato-κ*O*)bis­(nicotinamide-κ*N* ^1^)cobalt(II)

**DOI:** 10.1107/S1600536813004984

**Published:** 2013-02-28

**Authors:** Öznur Dincel, Barış Tercan, Füreya Elif Öztürkkan, Hacali Necefoğlu, Tuncer Hökelek

**Affiliations:** a Department of Physics, Karabük University, 78050 Karabük, Turkey; b Department of Chemistry, Kafkas University, 36100 Kars, Turkey; c Department of Physics, Hacettepe University, 06800 Beytepe, Ankara, Turkey

## Abstract

In the title complex, [Co(C_7_H_4_ClO_2_)_2_(C_6_H_6_N_2_O)_2_(H_2_O)_2_], the Co^II^ cation is located on an inversion center and is coord­inated by two 2-chloro­benzoate anions, two nicotin­amide (NA) ligands and two water mol­ecules. The four O atoms in the equatorial plane around the Co^II^ cation form a slightly distorted square-planar arrangement, while the slightly distorted octa­hedral coordination is completed by the two pyridine N atoms of the NA ligands in the axial positions. The dihedral angle between the carboxyl­ate group and the adjacent benzene ring is 29.7 (4)°, while the pyridine and benzene rings are oriented at a dihedral angle of 83.17 (15)°. Intra­molecular O—H⋯O hydrogen bonding occurs between the carboxyl­ate group and coordinating water mol­ecule. In the crystal, inter­molecular N—H⋯O, O—H⋯O and weak C—H⋯O hydrogen bonds link the mol­ecules into a three-dimensional network.

## Related literature

For background to niacin, see: Krishnamachari (1974 ▶). For information on the nicotinic acid derivative *N*,*N*-diethyl­nicotinamide, see: Bigoli *et al.* (1972 ▶). For related structures, see: Hökelek *et al.* (1996 ▶, 2009**a* ▶,b* ▶); Hökelek & Necefoğlu (1998 ▶, 2007 ▶); Necefoğlu *et al.* (2011**a* ▶,b* ▶). For bond-length data, see: Allen *et al.* (1987 ▶).
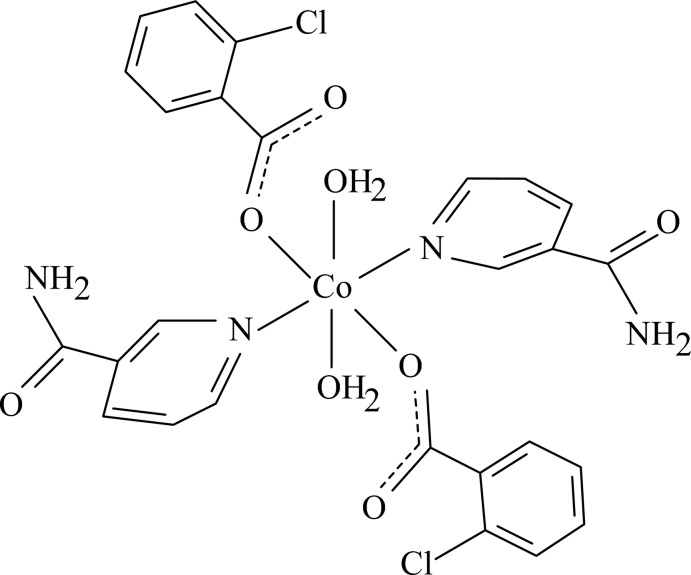


## Experimental

### 

#### Crystal data

[Co(C_7_H_4_ClO_2_)_2_(C_6_H_6_N_2_O)_2_(H_2_O)_2_]
*M* *_r_* = 650.32
Monoclinic, 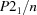

*a* = 7.8679 (2) Å
*b* = 17.9522 (3) Å
*c* = 9.8492 (2) Å
β = 106.637 (3)°
*V* = 1332.92 (5) Å^3^
*Z* = 2
Mo *K*α radiation
μ = 0.90 mm^−1^
*T* = 100 K
0.39 × 0.33 × 0.23 mm

#### Data collection

Bruker Kappa APEXII CCD area-detector diffractometer
Absorption correction: multi-scan (*SADABS*; Bruker, 2005 ▶) *T*_min_ = 0.708, *T*_max_ = 0.811
10778 measured reflections
3285 independent reflections
2366 reflections with *I* > 2σ(*I*)
*R*_int_ = 0.061

#### Refinement

*R*[*F* ^2^ > 2σ(*F* ^2^)] = 0.088
*wR*(*F* ^2^) = 0.237
*S* = 1.23
3285 reflections
202 parameters
H atoms treated by a mixture of independent and constrained refinement
Δρ_max_ = 1.42 e Å^−3^
Δρ_min_ = −1.61 e Å^−3^

### 

Data collection: *APEX2* (Bruker, 2007 ▶); cell refinement: *SAINT* (Bruker, 2007 ▶); data reduction: *SAINT*; program(s) used to solve structure: *SHELXS97* (Sheldrick, 2008 ▶); program(s) used to refine structure: *SHELXL97* (Sheldrick, 2008 ▶); molecular graphics: *ORTEP-3 for Windows* (Farrugia, 2012 ▶) and *PLATON* (Spek, 2009 ▶); software used to prepare material for publication: *WinGX* (Farrugia, 2012 ▶) and *PLATON*.

## Supplementary Material

Crystal structure: contains datablock(s) I, global. DOI: 10.1107/S1600536813004984/xu5678sup1.cif

Structure factors: contains datablock(s) I. DOI: 10.1107/S1600536813004984/xu5678Isup2.hkl

Additional supplementary materials:  crystallographic information; 3D view; checkCIF report

## Figures and Tables

**Table 1:** Selected bond lengths (Å)

Co1—N1	2.129 (4)
Co1—O2	2.102 (4)
Co1—O4	2.153 (4)

**Table 2:** Hydrogen-bond geometry (Å, °)

*D*—H⋯*A*	*D*—H	H⋯*A*	*D*⋯*A*	*D*—H⋯*A*
N2—H21⋯O1^i^	0.82 (10)	2.11 (9)	2.861 (6)	152 (9)
N2—H22⋯O3^ii^	0.90 (7)	2.20 (6)	2.932 (7)	138 (5)
O4—H41⋯O3^iii^	0.78 (9)	2.20 (10)	2.890 (6)	147 (10)
O4—H42⋯O1	0.96 (9)	1.72 (9)	2.628 (7)	155 (8)
C6—H6⋯O1^iv^	0.93	2.55	3.468 (8)	170
C10—H10⋯O1^i^	0.93	2.60	3.476 (7)	158

Symmetry codes: (i) 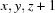; (ii) 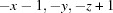; (iii) 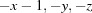; (iv) 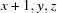.

**Fig. 1.**
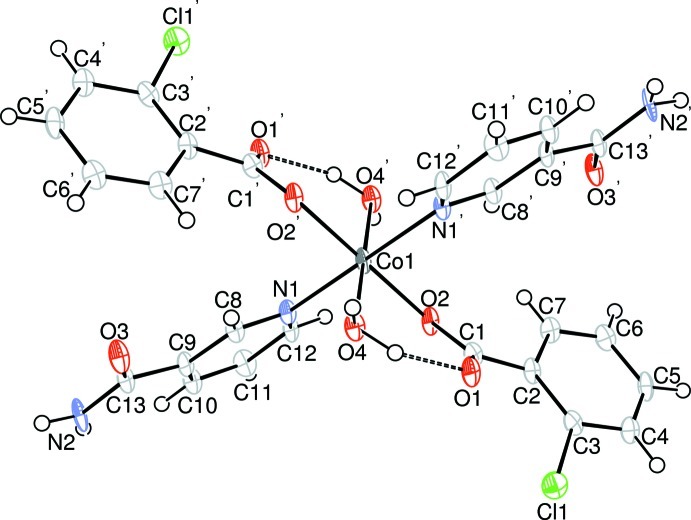
The molecular structure of the title compound with the atom-numbering scheme. Displacement ellipsoids are drawn at the 50% probability level. Primed atoms are generated by the symmetry operator: (') 1-x, -y, -z.

## supplementary crystallographic information

### Comment

As a part of our ongoing investigation on transition metal complexes of nicotinamide (NA), one form of niacin (Krishnamachari, 1974), and/or the nicotinic acid derivative *N*,*N*-diethylnicotinamide (DENA), an important respiratory stimulant (Bigoli *et al.*, 1972), the title compound was synthesized and its crystal structure is reported herein.

The title compound, (I), is a mononuclear complex, where the Co^II^ ion is located on a crystallographic inversion center. The asymmetric unit contains one 2-chlorobenzoate (CB) anion, one nicotinamide (NA) ligand and one coordinated water molecule, all ligands are monodentate (Fig. 1). The crystal structures of similar complexes of Cu^II^, Co^II^, Ni^II^, Mn^II^ and Zn^II^ ions, [Cu(C_7_H_5_O_2_)_2_(C_10_H_14_N_2_O)_2_] (Hökelek *et al.*, 1996), [Cu(C_7_H_4_BrO_2_)_2_(C_6_H_6_N_2_O)_2_(H_2_O)_2_] (Necefoğlu *et al.*, 2011*a*), [Co(C_6_H_6_N_2_O)_2_(C_7_H_4_NO_4_)_2_(H_2_O)_2_] (Hökelek & Necefoğlu, 1998), [Co(C_9_H_9_O_2_)_2_(C_10_H_14_N_2_O)_2_(H_2_O)_2_] (Necefoğlu *et al.*, 2011*b*), [Ni(C_7_H_4_ClO_2_)_2_(C_6_H_6_N_2_O)_2_(H_2_O)_2_] (Hökelek *et al.*, 2009*a*), [Mn(C_9_H_10_NO_2_)_2_(H_2_O)_4_].2H_2_O (Hökelek & Necefoğlu, 2007) and [Zn(C_7_H_4_BrO_2_)_2_(C_6_H_6_N_2_O)_2_(H_2_O)_2_] (Hökelek *et al.*, 2009*b*) have also been reported. In the copper(II) complex mentioned above the two benzoate ions coordinate to the Cu^II^ atom as bidentate ligands, while in the other structures all the ligands coordinate in a monodentate manner.

In the title complex, the four symmetry related O atoms (O2, O2', O4 and O4') in the equatorial plane around the Co^II^ ion form a slightly distorted square-planar arrangement, while the slightly distorted octahedral coordination is completed by the two symmetry related N atoms of the NA ligands (N1 and N1') in the axial positions (Fig. 1).

The near equalities of the C1—O1 [1.254 (7) Å] and C1—O2 [1.273 (7) Å] bonds in the carboxylate group indicates a delocalized bonding arrangement, rather than localized single and double bonds. The Co—O bond lengths are 2.102 (4) Å (for benzoate oxygens) and 2.153 (4) Å (for water oxygens), and the Co—N bond length is 2.129 (4) Å, close to standard values (Allen *et al.*, 1987). The Co atom is displaced out of the least-squares plane of the carboxylate group (O1/C1/O2) by -0.6474 (1) Å. The dihedral angle between the planar carboxylate group and the adjacent benzene ring A (C2—C7) is 29.65 (41)°, while that between rings A and B (N1/C8—C12) is 83.17 (15)°. Intramolecular O—H···O hydrogen bonding occurs between the carboxylate group and coordinated water molecule (Table 1, Fig. 1).

In the crystal structure, intermolecular N—H···O, O—H···O and weak C—H···O hydrogen bonds (Table 1) link the molecules into a three-dimensional supramolecular network.

### Experimental

The title compound was prepared by the reaction of CoSO_4_.7H_2_O (1.40 g, 5 mmol) in H_2_O (40 ml) and nicotinamide (1.78 g, 10 mmol) in H_2_O (20 ml) with sodium 2-chlorobenzoate (2.23 g, 10 mmol) in H_2_O (50 ml). The mixture was filtered and set aside to crystallize at ambient temperature for two weeks, giving pink single crystals (yield; 3.065 g, 72%).

### Refinement

Atoms H21, H22 (for NH_2_) and H41, H42 (for H_2_O) were located in a difference Fourier map and refined isotropically. The C-bound H-atoms were positioned geometrically with C—H = 0.93 Å for aromatic H atoms and constrained to ride on their parent atoms, with *U*_iso_(H) = 1.2*U*_eq_(C). The highest residual electron density was found 1.17 Å from Co1 and the deepest hole 0.80 Å from Co1.

### Crystal data

**Table:**

[Co(C_7_H_4_ClO_2_)_2_(C_6_H_6_N_2_O)_2_(H_2_O)_2_]	*F*(000) = 666
*M**_r_* = 650.32	*D*_x_ = 1.620 Mg m^−^^3^
Monoclinic, *P*2_1_/*n*	Mo *K*α radiation, λ = 0.71073 Å
Hall symbol: -P 2yn	Cell parameters from 4735 reflections
*a* = 7.8679 (2) Å	θ = 2.4–27.9°
*b* = 17.9522 (3) Å	µ = 0.90 mm^−^^1^
*c* = 9.8492 (2) Å	*T* = 100 K
β = 106.637 (3)°	Block, pink
*V* = 1332.92 (5) Å^3^	0.39 × 0.33 × 0.23 mm
*Z* = 2	

### Data collection

**Table:**

Bruker Kappa APEXII CCD area-detector diffractometer	3285 independent reflections
Radiation source: fine-focus sealed tube	2366 reflections with *I* > 2σ(*I*)
Graphite monochromator	*R*_int_ = 0.061
φ and ω scans	θ_max_ = 28.3°, θ_min_ = 2.3°
Absorption correction: multi-scan (*SADABS*; Bruker, 2005)	*h* = −6→10
*T*_min_ = 0.708, *T*_max_ = 0.811	*k* = −23→18
10778 measured reflections	*l* = −13→12

### Refinement

**Table:**

Refinement on *F*^2^	Primary atom site location: structure-invariant direct methods
Least-squares matrix: full	Secondary atom site location: difference Fourier map
*R*[*F*^2^ > 2σ(*F*^2^)] = 0.088	Hydrogen site location: inferred from neighbouring sites
*wR*(*F*^2^) = 0.237	H atoms treated by a mixture of independent and constrained refinement
*S* = 1.23	*w* = 1/[σ^2^(*F*_o_^2^) + (0.0784*P*)^2^ + 7.9389*P*] where *P* = (*F*_o_^2^ + 2*F*_c_^2^)/3
3285 reflections	(Δ/σ)_max_ < 0.001
202 parameters	Δρ_max_ = 1.42 e Å^−^^3^
0 restraints	Δρ_min_ = −1.61 e Å^−^^3^

### Special details

**Table:**

Geometry. All esds (except the esd in the dihedral angle between two l.s. planes) are estimated using the full covariance matrix. The cell esds are taken into account individually in the estimation of esds in distances, angles and torsion angles; correlations between esds in cell parameters are only used when they are defined by crystal symmetry. An approximate (isotropic) treatment of cell esds is used for estimating esds involving l.s. planes.
Refinement. Refinement of F^2^ against ALL reflections. The weighted R-factor wR and goodness of fit S are based on F^2^, conventional R-factors R are based on F, with F set to zero for negative F^2^. The threshold expression of F^2^ > 2sigma(F^2^) is used only for calculating R-factors(gt) etc. and is not relevant to the choice of reflections for refinement. R-factors based on F^2^ are statistically about twice as large as those based on F, and R- factors based on ALL data will be even larger.

### Fractional atomic coordinates and isotropic or equivalent isotropic displacement parameters (Å^2^)

**Table:**

	*x*	*y*	*z*	*U*_iso_*/*U*_eq_	
Co1	0.0000	0.0000	0.0000	0.0169 (3)	
Cl1	−0.0922 (2)	0.28811 (9)	−0.32577 (17)	0.0282 (4)	
O1	−0.1130 (5)	0.1367 (2)	−0.2254 (4)	0.0223 (9)	
O2	0.1155 (5)	0.0988 (2)	−0.0461 (4)	0.0192 (8)	
O3	−0.4515 (5)	0.0095 (3)	0.3249 (4)	0.0250 (10)	
O4	−0.2634 (6)	0.0436 (3)	−0.0890 (4)	0.0211 (9)	
H41	−0.329 (12)	0.014 (5)	−0.133 (10)	0.04 (3)*	
H42	−0.241 (11)	0.084 (5)	−0.146 (9)	0.05 (2)*	
N1	−0.0041 (6)	0.0446 (3)	0.1993 (5)	0.0175 (10)	
N2	−0.3567 (7)	0.0816 (3)	0.5201 (5)	0.0244 (12)	
H21	−0.282 (12)	0.108 (5)	0.573 (10)	0.05 (3)*	
H22	−0.457 (8)	0.071 (3)	0.543 (7)	0.013 (15)*	
C1	0.0477 (7)	0.1383 (3)	−0.1558 (6)	0.0180 (11)	
C2	0.1707 (8)	0.1857 (3)	−0.2106 (6)	0.0188 (11)	
C3	0.1181 (8)	0.2488 (3)	−0.2961 (6)	0.0196 (11)	
C4	0.2310 (8)	0.2848 (4)	−0.3596 (6)	0.0214 (12)	
H4	0.1919	0.3263	−0.4166	0.026*	
C5	0.4023 (8)	0.2589 (4)	−0.3380 (6)	0.0236 (12)	
H5	0.4774	0.2818	−0.3830	0.028*	
C6	0.4619 (8)	0.1984 (4)	−0.2485 (6)	0.0233 (12)	
H6	0.5784	0.1821	−0.2303	0.028*	
C7	0.3468 (8)	0.1629 (4)	−0.1870 (6)	0.0207 (12)	
H7	0.3879	0.1224	−0.1278	0.025*	
C8	−0.1512 (7)	0.0371 (3)	0.2405 (5)	0.0179 (11)	
H8	−0.2469	0.0113	0.1819	0.021*	
C9	−0.1672 (7)	0.0661 (3)	0.3663 (5)	0.0173 (11)	
C10	−0.0260 (8)	0.1057 (3)	0.4526 (6)	0.0211 (12)	
H10	−0.0339	0.1267	0.5369	0.025*	
C11	0.1259 (8)	0.1134 (4)	0.4115 (6)	0.0227 (12)	
H11	0.2225	0.1395	0.4680	0.027*	
C12	0.1337 (7)	0.0817 (3)	0.2846 (5)	0.0189 (11)	
H12	0.2375	0.0863	0.2581	0.023*	
C13	−0.3372 (7)	0.0508 (3)	0.4019 (6)	0.0188 (12)	

### Atomic displacement parameters (Å^2^)

**Table:**

	*U*^11^	*U*^22^	*U*^33^	*U*^12^	*U*^13^	*U*^23^
Co1	0.0160 (5)	0.0304 (6)	0.0024 (4)	−0.0006 (4)	0.000	−0.0003 (4)
Cl1	0.0217 (7)	0.0377 (9)	0.0220 (8)	0.0073 (6)	0.0009 (6)	0.0057 (6)
O1	0.019 (2)	0.039 (3)	0.0062 (18)	−0.0013 (17)	−0.0009 (15)	0.0015 (17)
O2	0.0159 (19)	0.035 (2)	0.0026 (17)	−0.0021 (16)	−0.0033 (15)	0.0011 (15)
O3	0.019 (2)	0.047 (3)	0.0072 (18)	−0.0064 (18)	0.0008 (15)	−0.0044 (18)
O4	0.020 (2)	0.034 (3)	0.0059 (19)	−0.0020 (18)	−0.0014 (16)	−0.0009 (18)
N1	0.018 (2)	0.029 (3)	0.004 (2)	−0.0013 (19)	−0.0005 (17)	−0.0005 (18)
N2	0.019 (3)	0.049 (4)	0.007 (2)	−0.007 (2)	0.007 (2)	−0.006 (2)
C1	0.017 (2)	0.027 (3)	0.007 (2)	−0.001 (2)	−0.001 (2)	−0.003 (2)
C2	0.020 (3)	0.029 (3)	0.004 (2)	−0.004 (2)	−0.001 (2)	−0.003 (2)
C3	0.018 (3)	0.031 (3)	0.007 (2)	0.001 (2)	−0.001 (2)	−0.004 (2)
C4	0.021 (3)	0.032 (3)	0.007 (3)	−0.003 (2)	−0.002 (2)	−0.001 (2)
C5	0.024 (3)	0.038 (3)	0.008 (3)	−0.007 (3)	0.004 (2)	−0.002 (2)
C6	0.019 (3)	0.034 (3)	0.015 (3)	−0.002 (2)	0.001 (2)	−0.004 (2)
C7	0.018 (3)	0.031 (3)	0.008 (2)	−0.002 (2)	−0.004 (2)	−0.002 (2)
C8	0.016 (3)	0.030 (3)	0.004 (2)	−0.002 (2)	−0.002 (2)	−0.002 (2)
C9	0.014 (2)	0.030 (3)	0.006 (2)	0.002 (2)	0.001 (2)	0.002 (2)
C10	0.023 (3)	0.032 (3)	0.006 (2)	−0.003 (2)	0.001 (2)	−0.004 (2)
C11	0.018 (3)	0.036 (3)	0.008 (3)	−0.003 (2)	−0.006 (2)	−0.005 (2)
C12	0.015 (3)	0.035 (3)	0.005 (2)	0.000 (2)	0.000 (2)	0.002 (2)
C13	0.013 (2)	0.036 (3)	0.006 (2)	0.000 (2)	0.000 (2)	0.001 (2)

### Geometric parameters (Å, º)

**Table:**

Co1—N1	2.129 (4)	C2—C7	1.399 (8)
Co1—N1^i^	2.129 (4)	C3—C4	1.384 (8)
Co1—O2	2.102 (4)	C4—C5	1.383 (8)
Co1—O2^i^	2.102 (4)	C4—H4	0.9300
Co1—O4	2.153 (4)	C5—H5	0.9300
Co1—O4^i^	2.153 (4)	C6—C5	1.392 (9)
Cl1—C3	1.744 (6)	C6—H6	0.9300
O1—C1	1.254 (7)	C7—C6	1.381 (8)
O2—C1	1.273 (7)	C7—H7	0.9300
O3—C13	1.243 (7)	C8—H8	0.9300
O4—H41	0.78 (9)	C9—C8	1.382 (7)
O4—H42	0.96 (9)	C9—C10	1.386 (8)
N1—C8	1.339 (7)	C9—C13	1.502 (7)
N1—C12	1.343 (7)	C10—C11	1.374 (8)
N2—C13	1.337 (7)	C10—H10	0.9300
N2—H21	0.81 (9)	C11—H11	0.9300
N2—H22	0.90 (6)	C12—C11	1.390 (8)
C1—C2	1.501 (8)	C12—H12	0.9300
C2—C3	1.402 (8)		
			
O2—Co1—O2^i^	180.0 (2)	C4—C3—C2	122.1 (5)
O2—Co1—O4	91.75 (17)	C4—C3—Cl1	115.9 (5)
O2^i^—Co1—O4	88.25 (17)	C3—C4—H4	120.1
O2—Co1—O4^i^	88.25 (17)	C5—C4—C3	119.8 (6)
O2^i^—Co1—O4^i^	91.75 (17)	C5—C4—H4	120.1
O2—Co1—N1	90.19 (16)	C4—C5—C6	119.7 (5)
O2^i^—Co1—N1	89.81 (16)	C4—C5—H5	120.1
O2—Co1—N1^i^	89.81 (16)	C6—C5—H5	120.1
O2^i^—Co1—N1^i^	90.19 (16)	C5—C6—H6	120.2
O4—Co1—O4^i^	180.0 (2)	C7—C6—C5	119.6 (6)
N1—Co1—O4	88.39 (17)	C7—C6—H6	120.2
N1^i^—Co1—N1	180.0 (3)	C2—C7—H7	118.8
N1^i^—Co1—O4	91.61 (17)	C6—C7—C2	122.3 (6)
N1—Co1—O4^i^	91.61 (17)	C6—C7—H7	118.8
N1^i^—Co1—O4^i^	88.39 (17)	N1—C8—C9	122.8 (5)
C1—O2—Co1	123.4 (4)	N1—C8—H8	118.6
Co1—O4—H41	113 (6)	C9—C8—H8	118.6
Co1—O4—H42	101 (5)	C8—C9—C10	118.7 (5)
H41—O4—H42	113 (8)	C8—C9—C13	117.1 (5)
C8—N1—Co1	119.0 (4)	C10—C9—C13	124.1 (5)
C8—N1—C12	118.3 (5)	C9—C10—H10	120.6
C12—N1—Co1	122.8 (4)	C11—C10—C9	118.9 (5)
C13—N2—H21	124 (6)	C11—C10—H10	120.6
C13—N2—H22	117 (4)	C10—C11—C12	119.3 (5)
H22—N2—H21	119 (7)	C10—C11—H11	120.4
O1—C1—O2	124.2 (5)	C12—C11—H11	120.4
O1—C1—C2	118.0 (5)	N1—C12—C11	122.0 (5)
O2—C1—C2	117.6 (5)	N1—C12—H12	119.0
C3—C2—C1	124.4 (5)	C11—C12—H12	119.0
C7—C2—C1	119.0 (5)	O3—C13—N2	122.3 (5)
C7—C2—C3	116.4 (5)	O3—C13—C9	120.2 (5)
C2—C3—Cl1	122.0 (4)	N2—C13—C9	117.4 (5)
			
O4—Co1—O2—C1	−34.9 (4)	C1—C2—C3—Cl1	10.4 (8)
O4^i^—Co1—O2—C1	145.1 (4)	C1—C2—C3—C4	−171.1 (5)
N1—Co1—O2—C1	−123.3 (4)	C7—C2—C3—Cl1	−175.5 (4)
N1^i^—Co1—O2—C1	56.7 (4)	C7—C2—C3—C4	3.0 (8)
O2—Co1—N1—C8	134.5 (4)	C1—C2—C7—C6	171.9 (5)
O2^i^—Co1—N1—C8	−45.5 (4)	C3—C2—C7—C6	−2.5 (8)
O2—Co1—N1—C12	−43.9 (4)	Cl1—C3—C4—C5	177.9 (4)
O2^i^—Co1—N1—C12	136.1 (4)	C2—C3—C4—C5	−0.7 (9)
O4—Co1—N1—C8	42.8 (4)	C3—C4—C5—C6	−2.3 (9)
O4^i^—Co1—N1—C8	−137.2 (4)	C7—C6—C5—C4	2.7 (9)
O4—Co1—N1—C12	−135.6 (5)	C2—C7—C6—C5	−0.3 (9)
O4^i^—Co1—N1—C12	44.4 (5)	C10—C9—C8—N1	1.1 (9)
Co1—O2—C1—O1	21.7 (8)	C13—C9—C8—N1	−177.6 (5)
Co1—O2—C1—C2	−154.0 (4)	C8—C9—C10—C11	−1.3 (9)
Co1—N1—C8—C9	−178.2 (4)	C13—C9—C10—C11	177.2 (6)
C12—N1—C8—C9	0.3 (9)	C8—C9—C13—O3	4.8 (8)
Co1—N1—C12—C11	177.0 (4)	C8—C9—C13—N2	−176.9 (6)
C8—N1—C12—C11	−1.4 (8)	C10—C9—C13—O3	−173.8 (6)
O1—C1—C2—C3	26.3 (8)	C10—C9—C13—N2	4.5 (9)
O1—C1—C2—C7	−147.6 (5)	C9—C10—C11—C12	0.3 (9)
O2—C1—C2—C3	−157.8 (5)	N1—C12—C11—C10	1.1 (9)
O2—C1—C2—C7	28.3 (8)		

Symmetry code: (i) −*x*, −*y*, −*z*.

### Hydrogen-bond geometry (Å, º)

**Table:**

*D*—H···*A*	*D*—H	H···*A*	*D*···*A*	*D*—H···*A*
N2—H21···O1^ii^	0.82 (10)	2.11 (9)	2.861 (6)	152 (9)
N2—H22···O3^iii^	0.90 (7)	2.20 (6)	2.932 (7)	138 (5)
O4—H41···O3^iv^	0.78 (9)	2.20 (10)	2.890 (6)	147 (10)
O4—H42···O1	0.96 (9)	1.72 (9)	2.628 (7)	155 (8)
C6—H6···O1^v^	0.93	2.55	3.468 (8)	170
C10—H10···O1^ii^	0.93	2.60	3.476 (7)	158

Symmetry codes: (ii) *x*, *y*, *z*+1; (iii) −*x*−1, −*y*, −*z*+1; (iv) −*x*−1, −*y*, −*z*; (v) *x*+1, *y*, *z*.
